# T cell immunotherapy for cervical cancer: challenges and opportunities

**DOI:** 10.3389/fimmu.2023.1105265

**Published:** 2023-04-26

**Authors:** Lingfeng Yu, Gong Lanqing, Ziyu Huang, Xiaoyan Xin, Liang Minglin, Lv Fa-hui, Hongmei Zou, Jie Min

**Affiliations:** ^1^ School of Basic Medical Sciences, Tianjin Medical University, Tianjin, China; ^2^ Department of Obstetrics and Gynecology, Union Hospital, Tongji Medical College, Huazhong University of Science and Technology, Wuhan, China; ^3^ School of Arts and Sciences, Brandeis University, Boston, MA, United States; ^4^ Department of Obstetrics and Gynecology, The Second People’s Hospital of Hefei, Hefei, Anhui, China; ^5^ Department of Obstetrics, Qianjiang Central Hospital, Qianjiang, Hubei, China

**Keywords:** cervical cancer, engineered T cells, TILs, TCR-Ts, CAR-Ts, immune checkpoint

## Abstract

Cancer cellular immunotherapy has made inspiring therapeutic effects in clinical practices, which brings new hope for the cure of cervical cancer. CD8+T cells are the effective cytotoxic effector cells against cancer in antitumor immunity, and T cells-based immunotherapy plays a crucial role in cellular immunotherapy. Tumor infiltrated Lymphocytes (TIL), the natural T cells, is approved for cervical cancer immunotherapy, and Engineered T cells therapy also has impressive progress. T cells with natural or engineered tumor antigen binding sites (CAR-T, TCR-T) are expanded *in vitro*, and re-infused back into the patients to eradicate tumor cells. This review summarizes the preclinical research and clinical applications of T cell-based immunotherapy for cervical cancer, and the challenges for cervical cancer immunotherapy.

## 1 Introduction

Cervical cancer is a disease in which malignant tumor cells are formed in the female cervix, and it is the most common malignant tumor in the female reproductive system. It occurs more commonly in women aged 40 to 60 years old than female of younger age groups.

At present, research on cervical cancer cannot fully explain the pathogenesis of cervical cancer yet, however, the evidence shows the onset of cervical cancer may be related to a series of factors such as early marriage, multiple births, cervical lacerations, poor local hygiene, and smegma irritation. Since the nineties, research has further confirmed that human papillomavirus (HPV) plays a significant role in the pathogenesis of cervical cancer (1).

It is found that almost all (99%) cases of cervical cancer, patients have persistent cervical HPV infection (2). There are more than 1,00 subtypes of HPV virus; among all, 40 subtypes are associated with reproductive tract diseases and 10 high-risk types are associated with cervical cancer. Particularly, infected by HPV-16 and/or HPV-18 would result in a higher risk of getting cervical cancer. Similar to HPV-16 and HPV-18, HPV-31 and HPV-33 infections also increase the risk of cervical cancer, yet are relatively less harmful (3).

It has been confirmed that persistent infection of HPV is a critical condition for cervical cancer occurrence. However, HPV infection alone cannot explain the abnormal cause of cervical epithelium, and infection alone is not enough to cause complete malignant changes in the cervical epithelium. More in-depth research on the process involved in the change and molecular mechanisms are needed.

Today, cervical cancer continues to have a high incidence in low- and middle-income countries, and nearly ninety percent of all cases of cervical cancers occurring in low- and middle-income countries are due to a lack of tissue screening and HPV vaccination for a variety of reasons (4, 5).

According to the different stages and specific conditions of the progress of cervical cancer, five therapies will be adopted in clinical practice including surgery, radiotherapy, chemotherapy, targeted therapy and immunotherapy. Among all, T-cell immunotherapy introduced in this article belongs to immunotherapy.

In recent years, the routine treatment of cervical cancer has progressed. Medical workers have tried to use new chemotherapy drugs and more effective radiotherapy methods and combined them to find more effective treatments.

For example, for locally advanced cervical cancer (International Federation of Gynecology and Obstetrics (FIGO) Phase IIB – IVA), platinum radiochemotherapy has been the standard treatment in the past two decades. There is evidence that it is superior to radiotherapy alone. However, this treatment has been plagued by the negative impact of disease recurrence and chemotherapy toxicity.

Several therapeutic plans that can help to change the clinical outcome of locally advanced cervical cancer have been studied. Gemcitabine, for example, is known to synergize with radiation and cisplatin. However, the improvement of patients’ quality of life achieved by new therapies is still limited by drug toxicity, for instance. Currently, the clinical guidance is more inclined to prevent cervical cancer from developing into a more malignant stage (6). For advanced cervical cancer, many studies have turned to palliative treatment to reduce the pain of patients, and to improve the quality of life of patients such as palliative surgery, analgesia drugs, or neurosurgery to prevent pain (6).Advanced cervical cancer can only be treated with chemotherapy or radiotherapy, but the prognosis is poor with the median survival rate of 16.8 months. For these patients, more effective treatment is urgently needed (7).

Among the traditional procedures for cervical cancer, surgery can directly eradicate cancer lesions, but there is a risk of recurrence, and the surgery is not effective when a cancer has metastasized. Radiotherapy and chemotherapy use the different sensitivity of cancer cells and normal cells to radiation and drugs to treat cancer. However, the similarity between cancer cells and normal cells makes a strong negative effect on normal tissues for both therapies. To prevent excessive injury, their application is greatly limited and cancer cells cannot be eradicated.

Targeted drugs can target specific types of cancer cells to achieve the goal of killing cancer cells without killing normal cells. Targeted drugs have played a revolutionary role in specific cancer treatments. However, only a small part of known cancer can be treated by targeted drugs. Inventing new cancer drugs is slow and costly, and the high cost also limits the application of targeted drugs.

As a new therapy, immunotherapy aims to make the human immune system respond more effectively to cancer cells. Immunotherapy includes immune checkpoint inhibitor,monoclonal antibody, vaccine, immunomodulator and T cell transfer therapy. Through *in vitro* editing, it cultivates T cells with stronger response ability to cancer cells and imports them into the body to treat cancer.

Adoptive T cell therapy (ACT) is a type of cancer immunotherapy that uses a patient’s immune cells to find and eliminate tumor cells, and donor immune cells can also be used in some cases (8). These include Chimeric Antigen Receptor T cells (CAR-T cells), T Cell Receptor-modified T cell (TCR-T) therapy, and Tumor-Infiltrating Lymphocyte (TIL) therapy.

Among them, TIL therapy belongs to natural T cell therapy, while CAR-T and TCR-T belong to engineering T cell therapy. The difference between TCR-T and CART-Ts is that CAR-T have weaker sensitivity and affinity to cancer cells. With MHC protein TCR-T can recognize proteins in cancer cells, whereas for CAR-T, CAR-T cells display target markers independently through MHC. Due to such characteristic, CAR-T cells need multiple targets to get triggered simultaneously to be effectitive. Compared to TCR-T cells, the affinity of CAR-T cells is more controllable, but CAR-T cells can only recognize proteins on the cancer cell surface but not those inside. In addition, there is evidence that the cytokine release syndrome caused by CAR-T cell therapy is more severe.

Some unconventional T cells and non-T cells, including NK, CD4+CD25+ or FoxP3+ Tregs, Th17 cells, γ-δT cells, and acrophages, have also been used in adoptive immunotherapy for cervical cancer.

NK cells are an important subset of TILs with emerging evidence indicateing their ability in killing cancer cells with a wide spectrum of cancer cells without the restriction of MHC match (9). An early study reported the detection of TIA-2, a molecule that reacts with the cytoplasmic domain of the zeta chain in CD3+ T and CD16+ NK cells. A marked decrease (p < 0.01) in expression of the CD3 ζ chain of PBLs in patients with cervical cancer (n = 22) as compared to PBLs from healthy donors (n = 21) was found. Moreover, PBLs isolated from patients (n = 23) with CIN, to a lesser but significant (p < 0. 01) extent expressed reduced CD3ζ levels as compared to those from healthy donors. This decreased expression of ζ chains was also observed on CD16+ NK cells in PBLs from patients with cervical cancer (10). These findings suggest that alterations of signal-transducing ζ molecules commonly occur in TILs, including NK cells of patients with cervical cancer, which may relate to their functionality and prognostic value.

Both CD4+CD25+ and FoxP3+ Tregs were reported in TILs of cervical cancer. They represent the opposite side of immunity by effectors and negatively regulate effector TIL functions. A study evaluated the FoxP3+ TILs in formalin-fixed paraffin-embedded tissues from 96 cervical cancer patients. The immunostaining density and other clinicopathological features such as FIGO stage, histopathologic type, Ki67 index, HPV status, lymphovasular invasion status, lymph node metastasis, tumor size, stromal invasion status, and parametrial invasion were evaluated for their roles in risk stratification of cervical cancer patients. The patients were stratified into low-, intermediate-, and high-risk groups, and a Spearman’s correlation analysis demonstrated that FoxP3+ TILs in the central tumor area showed a statistically negative correlation with risk stratification (p = 0.009) (11). It confirmed the prognostic value of Tregs in cervical cancer development.

Th17 is a minority cell population in TILs, there are few studies on Th17 cells. However, they may promote tumor progression by fostering angiogenesis and are therefore worth noting. A review article systemically summarized the roles of Th17 cells in cervical cancer and stated that the viral infection alone was not sufficient for the development and progression of premalignant cervical lesions to cancer. The hypothesis was that Th17 cells might be involved in the promotion of uterine cervical cancer (UCC), as high levels of IL-17 expression were detected in the mucosa of the uterine cervix of patients affected by the disease (12).

γ-δT cells is a special subset of T cells. In a study, γ-δT cells in combination with galectin-1 antibody treatment, significantly suppressed the growth of tumor xenografts in severe combined immunodeficiency (SCID) mice (p < 0.05), although γδ TILs alone showed the ability to inhibit tumor growth in vivo. Thus, the *in-vitro* expansion of this specific cell population may be necessary for their clinical application as TIL therapy (13).

TME plays essential roles in cancer development, and TAMs are a major player in the TME. A study investigated the density of TAMs in intraepithelial and tumor stromal areas of 148 patients with cervical adenocarcinoma, who were divided into two groups showing high and low cell infiltration, using the median value as a cutoff. Most cases (54.7%) were classified in stage IBb and 26 (17.6%) had recurrent disease. The density of stromal CD68+ or CD204+ macrophages that had infiltrated invasive adenocarcinoma (n = 127) was significantly higher than in adenocarcinoma *in situ* (n = 27). The Kaplan-Meier survival analyses revealed that a higher density of tumor-infiltrating CD204+ M2 macrophages was significantly associated with shorter disease-free survival (p = 0.0027) (14). Tumor-infiltrating CD204+ M2 macrophages are a prognostic factor for patients with cervical adenocarcinoma.

## 2 TILs and engineered T cells

### 2.1 TIL therapy

In the process of cancer, cancer cells can escape from \ the immune system, which is a necessary condition for tumor malignancy. However, many lymphocytes have detected abnormalities in tumors and infiltrated and existed in tumor tissues.These cells are tumor-infiltrating lymphocytes (TILs). They are characterized by more HPV specific T cells in TIL.

Although TILs do not play a sufficient role in the human body due to various immune escape mechanisms of cancer cells, they]identify multiple immune sites of cancer cells. Therefore, through screening, culture, and expansion of these immune cells, we can obtain many immune cells with strong aggressiveness to them.If it is infused back into the body, it can cure cancer.

In addition, gene editing can also enhance the ability of immune cells to recognize and kill cancer cells. At the same time, immunomodulators can be used to combat the inhibition of cancer cells on the immune system and enhance the therapeutic effect.

Prior to TIL therapy, there had been therapies to obtain immune cells from peripheral blood. However, experiments have proved that tumor infiltrating lymphocytes can produce better results (15). The advantage of TIL therapy for the following two other immunocellular therapies is that TILs naturally contain multiple sites to recognize cancer cells, while CAR-Ts and TCR-Ts need to be modified to adapt to a certain site. Therefore, TILs can prevent cancer cells from escaping from treatment through the mutation failure of a certain site (15).

At present, TIL therapy is primarily used for treating melanoma, and it has achieved effective results. In recent years, this therapy has been used for a variety of solid cancers, including cervical cancer, which will be introduced in this article and explored in more indications (16).

### 2.2 T cell receptor-modified T cell therapy

Tumor-specific T cell receptor T cell therapy is a therapy that enables T cells to kill cancer cells by transferring specific genes into T cells of patients to express specific receptors that can recognize tumor cells.

Tumor-specific T cell receptor is a kind of T cell receptor. T cell receptor is a key feature of T cells that distinguishes them from other lymphocytes. The tumor-specific T cell receptor can recognize specific antigen sites on the surface of tumor cells so that T cells can kill cancer cells.

By collecting naturally produced TCRs and selecting TCRs according to the characteristics of patients’ cancer cells and transferring them into T cells obtained from patients, screening and amplifying T cells that successfully express TCR, and then transferring them back to patients. Such a process shows to be effective in curing cancer.Because these TCRs are natural sources, they have a good ability to bind cancer cells.At the same time, TCR on TCR-T can recognize peptide complexes and human leukocyte antigens (HLA), which helps them distinguish normal cells in the human body from diseased cells, such as cancer cells. In this way, TCR-T will not affect normal tissues.

In most clinical trials, peripheral blood T cells for genetic modification are obtained *via* leukapheresis and are transduced by gamma-retroviral or lentiviral vectors that incorporate the TCR genes into the host genome, which results in high-level expression of the introduced TCR (17). Other means of genetic engineering that are currently in development include the transposon/transposase system, such as Sleeping Beauty (18), or Crispr/Cas9 based technology (19). These technologies do not require the production of lenti- or gamma-retroviral vectors and may therefore provide a more flexible and cheaper platform.

The difference between TCR-Ts and TIL therapy is that TIL therapy is based on TIL naturally existing in tumors, while TCR-T therapy is based on peripheral blood T cells isolated and genetically modified *in vitro* to express TCR targeting specific tumor antigens. And the difference between TCR-Ts and CART-Ts is that TCR-T have weaker sensitivity and affinity to cancer cells, and because the MHC they recognize can present proteins in cells, TCR-T can recognize proteins in cancer cells (20).

### 2.3 Chimeric antigen receptor T cell therapy

The treatment of chimeric antigen receptor T cells is similar to TCR-T, which is also transforming the patient’s T cells *in vitro* to become T cells with a specific ability to recognize cancer cells, and then expand and deliver them back to the patient.

The difference is that the chimeric antigen receptor (CAR) is an artificial hybrid receptor. At present, after genetic construction, scFv containing monoclonal antibody is used as antigen binding extracellular domain, and intracellular CD3ζ chain acts as a TCR signal domain and an additional co signal domain, mainly CD28 and 4-1BB (CD137) or others, to provide co-stimulation.

There are multiple ways to transfer the CAR genes into T cells. So far, the most common method is using retrovirus-infected cells. Evidence suggests that this method is effective and safe enough.

After nearly 30 years of development, CAR-T cells can be divided into four stages of development according to the different CARs: non-costimulatory signal CAR-T cells (the first generation), single-costimulatory signal CAR-T cells (the second generation), double/multiple costimulatory signal CAR-T cells (the third generation) and precise CAR-T cells (the fourth generation). The extracellular recognition region and transmembrane region of these four generations of CAR-T cells are not very different, so their stages are divided according to the structure of the intracellular segment of CAR.

In the first generation of CAR-T, the structure of CAR intracellular segment is relatively simple, mainly composed of the ITAM of CD3 molecule ζ Chain (21).

The second generation of CAR-T, based on the first generation of CAR-T cells, added an ITAM region from the costimulatory molecule CD28 or CD137 (4-1BB) in the intracellular segment. After the antigen recognition region outside the cell is combined with the target antigen, the T cells can obtain the antibody stimulation signal and the costimulatory signal at the same time (22).

The third generation CAR-T, the main structure of it is like that of the second-generation CAR-T cells, but the third generation CAR-T cells use lentivirus as the transfection vector, which can carry larger gene fragments into T lymphocytes. In theory, the third generation of CAR-T cells should have more activation and killing ability than the second generation of CAR-T cells (23).

The fourth generation CAR-T, also known as Precision CAR-T. The design of this generation of CAR-T cells is considered from the perspective of precise treatment of tumors and other diseases. Some studies have added suicide genes or controllable suicide genes (such as some drug-sensitive genes) to the structure of CAR, to control the survival time of CAR-T cells *in vivo* (24). Some studies have added the receptor structure of cytokine or chemokine in the design of CAR to increase the infiltration of T lymphocytes in tumor tissue, thus achieving the effect of enhancing the killing of solid tumors (25).

At the same time, there is also an emerging CAR-T called the fifth generation CAR-T, namely Ready-use CAR-T.

Ready-use CAR-T is a CAR-T prepared from allogenic T cells. Normally, T cells are collected from healthy donors or directly used from stem cells. After HLA matching, the designed CAR is loaded onto the surface of the T cells. The use of universal CAR-T therapy may provide a way to simplify the manufacturing of engineered cells and promote faster and cheaper treatment. But at the same time, it also has higher technical barriers and higher requirements for security. Therefore, there are few Ready-use CAR-T products entering the clinical development stage (26, 27).

The difference between CARs receptors and TCRs is that CAR-T cells display target markers independently through MHC. Due to such characteristic, CAR-T cells need multiple targets to get triggered simultaneously to play a role, but compared to TCR-T cells, the affinity of CAR-T cells is more controllable. In addition, CAR-T cells can only recognize proteins on the cancer cell surface but not those inside. In addition, there is evidence that the cytokine release syndrome caused by CAR-T cell therapy is more severe.

In addition, CAR-T cell therapy is currently mainly used for the treatment of blood tumors, but the treatment and research of solid tumors are still extremely limited, and the prospect is pessimistic (2).

## 3 Studies of engineered T cells in cervical cancer

### 3.1 E6 and E7 oncoproteins are the attractive therapeutic targets for ACT

Cervical cancer is one of the most common cancers among women, and the vast majority of cervical cancer (99%) is related to the persistent infection of HPV. Among them, 70% of cervical cancer can be confirmed to be caused by HPV type 16 and 18, and it is HPV infection that gives the common characteristics of cervical cancer cells: E6 and E7 oncoproteins.

After a virus invades human cells, it will control normal cell structure to synthesize a series of proteins according to its genetic information to replicate itself. E6 and E7 oncoproteins are closely related to cancer among the proteins synthesized after the HPV virus invades human cells, and the expression level of E6 and E7 is related to the type of cervical lesions that may eventually occur.

E6 and E7 oncoproteins are expressed in the early life of HPV and affect the normal physiological function of cells by affecting normal signal proteins in cells. E6 binds to host E6-related proteins with ubiquitin ligase activity and acts on the ubiquitination of p53, leading to its proteasome degradation. E7 (in carcinogenic HPV) is the main transforming protein. E7 competes for the binding of retinoblastoma protein (pRb) and releases the transcription factor E2F to activate its target, thus promoting the cell cycle forward (2, 28). This allows infected cells to escape from the cell checkpoint under influence, resulting in abnormal proliferation (29). Thus, the differentiated host keratinocytes are kept in a state conducive to the replication and expansion of the virus genome.

The experiment shows, all HPV can induce transient proliferation, but only strains 16 and 18 can immortalize cell lines *in vitro* (28). Combined with clinical evidence, it can be considered that E6 and E7 oncoproteins are the keys to the carcinogenicity of HPV16 and 18. Therefore, these proteins, which can be found in all cancer cells that cause cancer by this mechanism, have become highly promising targets for cervical cancer treatment (30).

Today, great progress has been made in therapies that target E6 and E7 oncoproteins in a variety of ways, including targeted vaccines, genome editing technology targeting, nucleic acid-based therapies, and genome editing using programmable nucleases, phytotherapy, and the various immune-targeted therapies described below (31).

### 3.2 The ongoing therapy and study about T cells therapy against HPV cervical cancer

According to the NCCN Guidelines, nowadays, no adoptive T-cell therapy has been included. Among immunotherapies, only pembrolizumab (for PD-L1-positive or MSI-H/dMMR tumors) and nivolumab (for PD-L1 positive tumors) are used as one of the combination therapy drugs for systematic therapy, and the following content will introduce the clinical research (32, 33).

#### 3.2.1 TIL

##### 3.2.1.1 Approval therapy

At present, TIL therapy is the only mature and approved clinical therapy for HPV cervical cancer. For example, in 2019, FDA approved autologous TIL immunotherapy LN-145 for the treatment of recurrent, metastatic, or persistent cervical cancer. Studies have shown that it can be used as a single therapy and combined with anti PD-1 immunosuppressant (34).

There are already many studies underway regarding LN-145 for the treatment of cervical cancer. The most success research to date is according to a report by ASCO 2019 NCT03108495. LN-145, another TIL product developed by Iovance, showed promising preliminary therapeutic results in 27 patients with advanced cervical cancer who had received at least one previous chemotherapy. In this study, an objective response rate of 44% was observed, including one full response and nine partial responses. In contrast, the objective response rate of second-line chemotherapy and immunotherapy approved by these patients was in the range of 4-14% (35).

Ongoing research NCT03108495 uses the autologous TIL manufacturing process originally developed by NCI to treat patients with recurrent, metastatic, or persistent cervical cancer. The cell transplantation treatment used in the study included patients receiving NMA lymphocyte depletion preparation protocol, then autologous TIL infusion, and then IL-2 protocol to further explore the application of LN-145. Another study on LN-145 in the treatment of head and neck squamous cell carcinoma is also in progress. NCT03083873 LN-145 demonstrated significant objective response rates (ORR) and disease control rates (DCRs) in the treatment of cervical cancer (35).

##### 3.2.1.2 Clinical trails

The research on cancer immunotherapy has a long history. Before the 1990s, related studies revealed the particularity of tumor-infiltrating lymphocytes (TILs), experiments have proved that they are special lymphocytes, compared to other sources of immune cells, TILs have a more prominent ability to inhibit the development of tumors (36, 37).

Today, the study of TIL has gone further, and the latest data from a series of studies including melanoma, cervical cancer, and breast cancer have further confirmed the advantages of TILs. Data shows that TILs contain more specific immune cells for cancer, and their amplification in patients can obtain more effective ACT. At present, the research and clinical application of TILs are steadily advancing, and it can be expected that TILs-based therapies will be recognized by more clinicians and accepted by patients and society (38).

It is worth mentioning that since TILs come from patients themselves, they contain more specific cell populations that specifically identify tumors, which made it easier for TILs returning to the tumor site to kill tumor cells. This personalized feature, compared with traditional ACT, has more advantages and is a major prospect of precision medicine (39–41).

In earlier studies, the use of TIL has focused on its impact on cancer prognosis (42). In recent years, there are also other clinical studies with certain results:

A study that ended in 2018 investigated the efficacy and possible negative effects of TIL therapy on epithelial tissue tumors, including cervical cancer. The results showed that of the 18 cervical cancer patients tested, 2 developed a complete response to cervical cancer atrophy, and 3 produced a partial response with 1 in stable condition and 12 had progression on the cancer. The non-cervical cancer group had a poor effect, with only 18.2% of patients having a partial response. At the same time, none of the patients tested experienced serious adverse reactions. NCT01585428

At present, more research on TIL therapy is being carried out extensively, which shows that the academic community attaches importance to this field: NCT05475847

A Phase I trial to evaluate the safety, tolerability, and initial antitumor activity of C-TIL052A cells in the treatment of persistent, recurrent, and/or metastatic cervical cancer. NCT04443296 A phase I trial to evaluate the feasibility, toxicity and effectiveness of cisplatin combined with chemotherapy and radiotherapy plus TIL in the treatment of patients with FIGO IIIA to IVA cervical cancer. Thus it is clear that the combination of TIL therapy and traditional therapy is also a widely concerned research direction.

Other trials include: NCT04674488 which studying advanced, spread cervical cancer; NCT05366478 To study the efficacy of advanced cervical cancer.

A phase II study (NCT01585428) of autologous tumor-infiltrating lymphocytes and aldesleukin for human papillomavirus-associated cancer, which ended observation in 2016, will diagnose patients with metastatic cervical cancer and who have previously received platinum-based chemotherapy or chemoradiotherapy for a single infusion of tumor-invasive T cells, these T Cells are selected for human papillomavirus (HPV) E6 and E7 reactivity (HPV-TILs) where possible. Cell infusion is preceded by lymphocyte-depleting chemotherapy, followed by aldesleukin administration. Three of the nine patients experienced objective tumor responses (two complete and one partial). The two complete responses continued 22 and 15 months after treatment, respectively. One partial response lasted 3 months. HPV reactivity of T cells in infusion products (as measured by interferon γ production, enzyme-linked immunospots, and CD137 upregulation assay) was positively correlated with clinical response (P = 0.0238 for all three assays). In addition, the frequency of HPV-reactive T cells in peripheral blood 1 month after treatment was positively correlated with clinical response (P = .0238).

Overall, the research on TIL in the treatment of cervical cancer is mainly based on phase I clinical trials, but it has shown quite a good prospect, and researchers are actively exploring. At the same time, (NCT03108495) as a recognized breakthrough therapy can be expected to be a bridge to higher practicality of TIL in the treatment of cervical cancer.

##### 3.2.1.3 Important studies in pre-clinical study

The principle of TIL treatment is to enhance and restore the anti-tumor immunity of TME by transferring cells from the immunosuppressive environment to the promoting environment, expanding *in vitro*, and reaching enough for infusion to patients. Therefore, an important goal of TIL research is to determine the composition of subtypes in the initial TIL population and prioritize the expansion (43).

The expression receptor of lymphocytes, as well as their changes and growth pattern, appears to be the focus of preclinical research on TIL therapy.

An early animal and clinical study indicated that the degree of tumor regression was significantly related to the extent of the presence of ACT cell clones in the body’s peripheral blood. This points out that the insufficient retention capacity of T cells may be one of the most crucial factors restricting the efficacy of TIL. The results showed that the telomere length of metastatic lymphocytes was related to the persistence of T cells *in vivo* after adoptive transfer (43–45).

Based on this research, young TIL therapy was developed in 2010, which is to gather lymphocytes from multiple tumor sites to obtain the number of cells required for rapid expansion. In this way, young TIL therapy shortened the culturing time, so that TILs with high CD27 and CD28 expression and long telomeres were enriched (46).

This rapid amplification method also brings significant benefits to clinical practice. Most importantly, it allows more patients to receive TIL treatment instead of missing the best treatment timing or does not meet the treatment condition due to the decreasing quality in TIL culturing process.

Conventional TIL therapy is generally stimulated by IL-2 *in vitro*, however, promoting the proliferation of TIL through other molecules is also a domain worth concerning. One study enhanced TIL production by targeting 4-1BB. 4-1BB is a costimulatory molecule on activated T cells, participating in T cell proliferation and antigen-specific cytolytic activity (47).

Other signal molecules include IL-15 and IL-21. Unlike IL-2, which promotes effector T cell differentiation and Treg proliferation and supports T cell activation-induced cell death (AICD), IL-15 and IL-21 induce younger, less differentiated central memory phenotypes and do not promote AICD. IL-2 promotes the differentiation of effector T cells and support the cell death induced by T cell activation (48, 49).

The antigen that can be recognized by T cells is not only derived from cell cancer. It is not the only source of tumor immunogenicity. Viral antigens are also an important source of tumor immunogenicity, so virus-infected cancers, such as those associated with the human tumor virus (HPV) or Epstein-Barr virus (EBV), can be triggered by T cell-mediated immunity (50).

In cervical cancer, most current studies focus on evaluating the prognostic values of TIL types and numbers in situ or ex vivo. Indeed, the number and composition of TILs reflect the process of the host immune system interacting with tumor cells and the TME, thus indicating cancer progress and treatment outcome. These TILs mainly consist of CD8+ and CD4+ T cells, NK cells, Tregs, and Th17 and γδ T cells. Some studies also included B cells and macrophages.

A study aims to gain insight into cervical tissue T cell populations, determine if there are any differences in the localization and quantity distribution of T lymphocytes, and to evaluate their role in disease regression or progression in the cervical neoplastic milieu. It analyzed the location and quantity of CD8+ and CD4+ TILs in the cervical neoplastic milieu of 72 samples using immunohistochemistry (IHC). The patients included four cohorts: 23 HPV non-infected (HPV−) normal cervix, 20 HPV-infected (HPV+) normal cervix, 17 HPV+ low-grade cervical intraepithelial neoplasia (CIN), and 12 HPV+ high-grade CIN. The results showed that a low level of TILs in normal cervix and an elevated level of TILs in CIN were present with a trend of TIL levels increasing with increases of the grade of CIN (p < 0.0001) (51). It is further supported by a similar study from 96 tissue section samples (including 26 CIN1, 21 CIN2, 25 CIN3, and 24 squamous cell carcinoma [SCC] samples) (52). Because HPV mainly infects epithelial cells, these data suggest that CD8+ TILs are at the frontline, fighting virus-infected cells as well as cancer cells.

A study compared lymphocytes in cervical tissues from 19 patients with pathologically confirmed CIN and from 20 patients with normal cervices undergoing hysterectomy for benign indications. The percentage of CD4+ T cells was significantly depressed (p = 0.04) in dysplastic tissue as compared to normal cervical tissue. In contrast, the proportion of CD8+ T cells was significantly increased in the dysplastic tissue (p = 0.0001) (53). Above data clearly indicate that the higher number and density of CD3+ and CD8+ TILs are positively associated with the progress of cervical cancer.

Although CD3+ TILs include a CD4+ subset, the direct evidence of CD4+ T cell association with cancer progress is not strong. Instead, a few studies reported that the decreased cell number of CD4+ T cells was associated with worse long-term survival rates of cervical cancer patients. For example, a study retrospectively analyzed 40 biopsy samples from Chinese cervical cancer patients and found, when considering the deaths and surviving cases as separate groups, that the number of CD4+ T cells was significantly lower in patients who died compared with those who survived (26.33 ± 11.80 versus 47.79 ± 38.18, p = 0.023) (1).

Clinical studies have shown that patients with HPV-positive head and neck squamous cell carcinoma who develop HPV-positive show increased responses against PD-1 and anti-PD-L1 inhibitors, pemulizumab, and dewarumab, respectively, compared with HPV-negative patients. In addition, increased efficacy of ICB was noted in patients with EBV and HIV-positive metastatic gastric cancer (15).

##### 3.2.1.4 Combination with immunocheckpoint

Immune checkpoints are molecules expressed on immune cells (such as T or B cells and APCs) that are used to negatively modulate the immune response (54). The existence and high expression of these molecules are important indicators of immune function inhibition. For cervical cancer, the expression of PD-1/PD-L1 is most related to HPV-related cancers, especially HPV16+and HPV18+cases. The reason is that the HPV E5/E6/E7 oncogene activates multiple signal pathways and finally regulates the PD-1/PD-L1 axis to promote HPV-induced cervical cancer. Both create opportunities for HPV infection and subsequent canceration by inhibiting immune function.

As a result, although tumor-infiltrating lymphocytes exist in tumors and can specifically identify tumors, they cannot inhibit the deterioration of cancer in the human body, because they are in an immunosuppressive environment. Although TIL therapy has expanded lymphocytes *in vitro*, these immune cells are still affected by the immunosuppression brought by the immune checkpoint, resulting in a great negative impact on the efficacy of TIL. If the checkpoint is suppressed before inputting TILs again, it will help TILs functions. At present, some researchers have used PD-1 inhibitors to assist TIL therapy to enhance the cytotoxicity of TILs. In addition, there are also attempts to silence the immune checkpoints by genetic engineering (55).

Immune checkpoint inhibitors (ICB) have long been an effective drug in solid tumor treatment and are most effective in melanoma treatment. According to an article in 2016, the response rate for combination therapy with anti-PD-1 and anti-CTLA4 for melanoma can reach 60% (56).

Immune checkpoint inhibitors (ICBs) have been used in cancer treatment for more than 10 years and are currently more practical than TIL ACT due to their widespread availability and availability. TIL ACT is a second-line treatment for patients who do not respond well to ICB therapy or who develop drug resistance. This approach has been shown to have objective and long-lasting effects, according to the literature, achieved in 20%-30% of patients (57–59).

For cervical cancer, a review article systemically analyzed 126 published papers and concluded that the expression of PD-1/PD-L1 was associated with HPV-related cancers, especially with HPV16+ and HPV18+ cases. The reason was that HPV E5/E6/E7 oncogenes activated multiple signaling pathways, including phosphatidylinositol 3-kinase (PI3K)/AKT, mitogen-activated protein kinase (MAPK), HIF1α, STAT3/nuclear factor κB (NF-κB), and microRNAs, which regulated the PD-1/PD-L1 axis to promote HPV-induced cervical carcinogenesis. The PD-1/PD-L1 axis then played a crucial role in immune escape of cervical cancer through inhibition of host immune response creating an “immune-privileged” site for initial viral infection and subsequent adaptive immune resistance (8). This analysis provides a rationale for therapeutic blockade of the PD-1/PD-L1 axis for HPV+ cancers.

A study investigated if immunotherapy against human papilloma virus (HPV) using a viral gene delivery platform to immunize against HPV 16 genes E6 and E7 (Ad5 [E1-, E2b-]-E6/E7) combined with programmed death-ligand 1 (PD-1) blockade could increase therapeutic effect as compared to the vaccine alone. Ad5 [E1-, E2b-]-E6/E7 as a single agent induced HPV-E6/E7 cell-mediated immunity. Immunotherapy using Ad5 [E1-, E2b-]-E6/E7 resulted in clearance of small tumors and an overall survival benefit in mice with larger established tumors. When immunotherapy was combined with immune checkpoint blockade, an increased level of anti-tumor activity against large tumors was observed. Analysis of the tumor microenvironment in Ad5 [E1-, E2b-]-E6/E7 treated mice revealed elevated CD8(+) tumor infiltrating lymphocytes (TILs); however, we observed induction of suppressive mechanisms such as programmed death-ligand 1 (PD-L1) expression on tumor cells and an increase in PD-1(+) TILs. When Ad5 [E1-, E2b-]-E6/E7 immunotherapy was combined with anti-PD-1 antibody, we observed CD8(+) TILs at the same level but a reduction in tumor PD-L1 expression on tumor cells and reduced PD-1(+) TILs providing a mechanism by which combination therapy favors a tumor clearance state and a rationale for pairing antigen-specific vaccines with checkpoint inhibitors in future clinical trials (60).

These support that inhibition of immune checkpoints such as PD-1 and its ligand PD-L1 may benefit immunotherapy of cervical cancer and the combination of TILs with ICB is a very promising research field.

#### 3.2.2 TCR-T

At present, TCR-T therapy has demonstrated clinical activity in melanoma and synovial cell sarcoma (61).

Consequently, the potential of TCR-T dramatically outweighs CAR-T in treating solid tumors. However, the utility of TCR-T in treating solid tumors is progressing slowly. Currently, there is no market approval for any TCR-T products. Several clinical trials are still ongoing (62).

One of the main factors limiting the use of TCR-T in cervical cancer is that because TCR-T acquires the ability to specifically recognize cancer cells through gene editing, researchers need to first look for effective targets that the corresponding cancer cells have. In turn, TCR-T cells can have the corresponding ability (63).

Therefore, the current research focuses on finding effective targets and testing their effectiveness in preclinical experiments. There are some promising results: in a study in 2018, researchers found HPV-16 E7 specific HLA-A * 02:01-restrictive TCR from cervical biopsy of a woman with cervical intraepithelial neoplasia. This TCR shows high functional affinity and has CD8 co receptor independent tumor targeting. TCR transduced human T cells specifically recognize and kill HPV-16+cervical cancer and oropharyngeal cancer cell lines and mediate the regression of established HPV-16+human cervical cancer tumors in a mouse model. These findings support the therapeutic potential of this approach (32).

##### 3.2.2.1 Clinical trails

So far, TCR based methods have shown clinical activity in the largest range of solid tumors. Compared with CAR, TCR’s antigen recognition system has the advantage that it can target peptides produced by intracellular or extracellular proteins (64).

The following are target antigens for engineered T cell therapy in solid cancers.

In a study that ended in 2017, the researchers conducted experiments on different administration methods for a total of 12 patients in groups, and only two of six patients in one group had partial reactions (partial reactions refer to the reduction of the sum of the longest diameter (LD) of target lesions by at least 30%). (NCT02280811)

Another study conducted a first-in-human, phase 1 clinical trial of T cells engineered with a T cell receptor targeting HPV-16 E7 for the treatment of metastatic human papilloma virus-associated epithelial cancers.The primary endpoint was maximum tolerated dose. Cell dose was not limited by toxicity with a maximum dose of 1×10^11^ engineered T cells administered. Tumor responses following treatment were evaluated using RECIST (Response Evaluation Criteria in Solid Tumors) guidelines. Robust tumor regression was observed with objective clinical responses in 6 of 12 patients, including 4 of 8 patients with anti-PD-1 refractory disease. Responses included extensive regression of bulky tumors and complete regression of most tumors in some patients. (NCT02858310)

More relevant research will be listed in the following table.

##### 3.2.2.2 Important studies in pre-clinical study

As mentioned earlier, E6 and E7 oncoproteins of HPV associated epithelial carcinoma are in principle ideal targets for immunotherapy. However, the evidence that T cells targeting these antigens can recognize and kill HPV (+) tumor cells is limited.

Similarly, TCR-T therapy also faces similar immunosuppression problems as TIL, so it also needs to be combined with corresponding immune checkpoint blockers in treatment.

A Phase I trial of HPV-16 E7-oncoprotein-targeted T cell receptor therapy, alone or in combination with the PD-1 inhibitor bloomivizumab, is currently recruiting HPV-associated cancer patients. (NCT02858310) Research on TCR-T therapy is also using emerging technologies. A study has established a mathematical model to study the efficacy of engineered T cell receptor (TCR) T cells targeting E7 antigen in treating cervical cancer cell lines (65). Another article reported the pre-clinical evaluation of a KK-LC-1 reactive T cell receptor (KK-LC-1 TCR), including *in vitro* tumor cell targeting, *in vivo* regression of xenograft tumors, cross-reactivity studies, and evaluation of the expression of healthy tissues and tumor antigens. This receptor derived from tumor-infiltrating lymphocytes of cervical cancer patients who have complete tumor response to TIL therapy (66).

In short, although the application of TCR-T therapy in cervical cancer is still far from clinical application, the prospects are still considerable.

Unlike TIL therapy, TCR-T therapy does not use immune checkpoint suppressors to enhance efficacy. In clinical trials for the treatment of melanoma, none of the TCR-T therapies used ICB. Such as (NCT00509288) (NCT00923195). More examples are in the **Table 1**.

**Table 1:** TCR-T therapy.

Type	antigen	Stage and Result	Host	NCT
TCR-T	HPV-E6	Phase I/II (completed with results)	National Institutes of Health Clinical Center, USA	NCT02280811
		One patient had SD for 6m, one had SD for 4m		
αPD1-TCR T	HPV-E6	Phase I (recruiting)	Qingzhu Jia, Chongqing, China	NCT03578406
		Enhanced SD in combination with anti-PD-1 therapy		
TCR-T	HPV-E7	Phase I/II (recruiting)	National Institutes of Health Clinical Center, USA	NCT02858310
TCR-T	HPV-E7	Early Phase I (suspended)	National Institutes of Health Clinical Center, USA	NCT04476251
TCR-T	HPV-E7	Phase I (withdrawn)	National Institutes of Health Clinical Center, USA	NCT04411134
TCR-CD4+ T	MAGE-A3	Phase I/II (active, not recruiting)	National Institutes of Health Clinical Center, USA	NCT02111850
		One patient had CR for > 29m		
TCR-T	MAGE-A3	Phase I/II (terminated)	National Institutes of Health Clinical Cente, USA	NCT02153905
		One patient had PR after 6w and 12w		

#### 3.2.3 CAR-T

##### 3.2.3.1 Approval therapy

Adoptive T cells, including chimeric antigen receptor T cell therapy, have been a cutting-edge approach to tumor treatment in recent years and are considered effective and promising. At present, CAR-T therapy is the most effective for hematological malignancies (67, 68). For example, there are many clinical trials using CAR-T cells targeting CD19 and BCMA, which have obtained encouraging clinical data in the treatment of B-ALL and multiple myeloma (69, 70).

However, CAR-T is still in the exploration stage in solid tumors. There is no CAR-T type therapy approved for clinical use (71). The limitation of the research is because of a lack of appropriate targets. On the other hand, it may also be that in solid tumors, compared with non-solid tumors, the tumor microenvironment will affect them (72, 73).

##### 3.2.3.2 Clinical research

An ideal antigen called CD19 exists in hematological tumors, however, there is no known antigen with similar characteristics in gynecologic cancers. At present, mesothelin, CA125, and folate receptors are the most widely studied antigens in these tumor CAR-T cell therapy trials. At present, the relevant clinical trials for the application of CAR-T in cervical cancer are limited (NCT01583686) (NCT04556669) (NCT03356795) and are among the few studies. Another prospective study involving cervical cancer is ongoing (NCT04556669). More examples are in the **Table 2**.

**Table 2:** CAR-T therapy.

Type	antigen	Stage and Result	Host	NCT
CAR-T	Mesothelin	Phase I (terminated)	National Institutes of Health Clinical Center, USA	NCT01583686
		Only one patient had SD for > 3.5m		
αPD1-CAR-T	CD22	Phase I (recruiting)	Fourth Hospital of Hebei Medical University, China	NCT04556669
CAR-T	GD2, PSMA, MUC1, Msln	Phase I/II (recruiting)	Shenzhen Geno-immune Medical Institute, China	NCT03356795

##### 3.2.3.3 Important studies in pre-clinical study

After some studies have confirmed that many genomic changes can be found in the cancer of CC patients, such as KRAS, PIK3CA, TP53, and PTEN. These mutations may be targeted as immunotherapy (74):

The application of CAR in cervical cancer and other solid tumors focuses on finding effective specific tumor antigens. A study published in 2022 tested placental alkaline phosphatase (PLAP). PLAP is a shared placental and tumor-associated antigen (TAA) that is expressed in ovarian, cervical, colorectal, and prostate cancers but rarely expressed in normal cells. The results show that PLAP CAR-T cells not only proliferate during co-culture with cancer cells but also remove them outside the body. The researchers also observed increased secretion of IL-2, granzyme A, and IFN-γ after PLAP CAR-T cells were exposed to target cells. Therefore, it was concluded that PLAP CAR-T cells are potential candidates for further study of cervical cancer and other solid tumors (75).

In another study, to improve antigenicity and reduce transformation activity, modified HPV16 E7 (HPV16ME7) was loaded with SOCS1 silenced dendritic cells (DC) to improve its efficiency and targeting against cervical cancer. The CAR-T-PD1 cells activated by the generated DC were injected into the CaSki cell tumor mouse model expressing PDL1 and HPV16 E6/E7 for *in vitro*/*in vivo* anti-tumor activity determination. The results showed that the gene engineering T cells activated by dendritic cells could improve the anti-tumor efficiency and targeting (76). In another study, the researchers designed an NKG2D CAR-T for NKG2DL. The results proved that NKG2D CAR-T has a highly effective anti-tumor ability against NKG2DL-positive cervical cancer cell lines *in vitro* (67).

The current research aims to find specific antigens suitable for CAR and to test various methods to improve the efficacy of CAR-T in solid tumors.

In terms of immune checkpoints, cervical cancer is known to frequently detect high levels of CTLA4 and PD1/PD-L1, which are often expressed in dendritic cells of cervical intraepithelial neoplasia (CIN) samples (77, 78). This may be the reason for the poor efficacy of CAR-T in cervical cancer, however, there is currently no evidence that immune checkpoint inhibitors are beneficial for CAR-T therapy.

The development of CAR technology in gynecological cancer is still in its early stages. A series of studies show that the current situation is far from satisfactory.

## 4 The challenges with engineered T cells in cervical cancer

Toxicity is a top priority for any new treatment, and it is as important as efficacy in early clinical studies and preclinical studies. One of the most striking features of adoptive T-cell therapy is its predictable low toxicity, while traditional radiotherapy and chemotherapy inevitably bring damage to the human body while treating cancer.

According to early clinical safety data, TIL therapy in ACT has a good safety profile, and in various experiments, side effects come from the regimen of combination administration with TIL therapy, such as IL-2 Simultaneous chemotherapy regimen (79). These toxicities will be observed eventually. Toxicity can be observed immediately or with delayed onset. Almost all patients receiving chemotherapy with nonmyeloablative lymphocytosis will experience hemocytopenia, including neutropenia, lymphocytopenia, and long-term inhibition of CD4 T cells (80–82). Significant toxicities associated with TIL therapy itself are rare, and these reactions are almost indistinguishable from those associated with residual IL-2 (83, 84).

In summary, TIL therapy has a variety of toxicities, most of which are low-grade and can be managed with standard supportive care (79).

The biggest problem with TIL therapy and even all ACT therapies is that it is a personalized medical plan. The cell injections that need to be imported into patients must be produced according to individual conditions, which leads to high costs, and more importantly, the production cycle is too long. As a result, when cancer progresses rapidly, ACT therapy cannot control cancer immediately. This limits the application scenarios of TIL therapy.

Today, there is also a lack of good methods and manufacturing practices for TIL production and automation. This makes this therapy more expensive and time-consuming.

For TCR-T therapy, the challenge is to find a suitable target. Some of the targets currently selected are TAAs, and although they do have elevated expression in cancer tissue, they are expressed at low levels in normal human tissue, so further research is needed to determine whether this condition leads to autoimmune toxicity or tolerance to engineered T cells.

Like the dilemma encountered by TIL therapy, because of the mutation of neoantigen formation is different for each patient, TCR-T treatment is customized for each patient, which makes it difficult to develop widely applicable immunotherapy products, resulting in a considerable cost related to TCR-T The longer manufacturing cycle and the more complex preparation method of TCR-T make this problem more serious than TIL.

In addition, while looking for new antigens that are widely shared in tumor cells, such as KRAS and TP53, are already being sought. However, in general, all aspects of research are still in a preliminary state (85, 86). At present, the prospect of CAR-T is quite worrying in all aspects. CAR technology lacks achievements in the field of solid tumors, and the reasons are not clear (87).

At the same time, CAR-T also showed more toxic reactions than other therapies, including anaphylaxis, graft versus host disease, nervous system toxicity (including confusion, delirium, aphasia, myoclonus, and seizures) and toxicity caused by missing target (88, 89).

## 5 The future of engineered T cells in the field of cervical cancer

At present, ACT in the treatment of cervical cancer, TILs technology is the most promising technology, which not only has LN-145 such as approved more mature technology, but also the corresponding clinical and preclinical research is also the most abundant. However, further specification of the production process is still required to improve production efficiency and safety.

For ACT therapy, in general, future research should focus more on:

(1) Advancing clinical and preclinical trials for already more mature therapies;
(2) Especially for TCR and CAR, it is necessary to find more suitable antigen-binding sites to strengthen their practicability;
(3) For existing therapies, the production speed should be accelerated, the supply should be increased, and safety should be held in terms of process and flow;
(4) Improve the activity of engineered T cells in the tumor microenvironment. Synergistic therapy with immunocheckpoint inhibitors or other substances can be the direction of exploration.
(5) Explore the synergistic possibilities of ACT in other cancer therapies available.

## 6 Summary

ACT therapy has brought new hope for the treatment of reproductive system cancer, including cervical cancer, especially TIL therapy, which has become a breakthrough therapy. TCR-T therapy also shows a promising future. Although CAR-T therapy seems to have less potential, its excellent performance in blood tumors still makes it have a promising future in the field of solid tumors, including cervical cancer.

However, ACT therapy also faces many challenges. The high cost caused by safety, preparation efficiency, individualization, etc. still restricts its wide application. The difficulties in selecting the appropriate antigen, immunosuppression, and short pharmacological duration also continue to perplex related research. At present, a series of clinical and preclinical studies based on different models are gradually exploring relevant issues, strengthening efficacy, reducing adverse reactions, and exploring the prospect of combined application with other therapies. ACT therapy has a promising future for the treatment of cervical cancer.

## Author contributions

All authors listed have made a substantial, direct, and intellectual contribution to the work and approved it for publication.
